# Nurses’ Contacts and Potential for Infectious Disease Transmission

**DOI:** 10.3201/eid1509.081475

**Published:** 2009-09

**Authors:** Helen Bernard, Richela Fischer, Rafael T. Mikolajczyk, Mirjam Kretzschmar, Manfred Wildner

**Affiliations:** Robert Koch Institute, Berlin, Germany (H. Bernard); Bavarian Health and Food Safety Authority, Oberschleissheim, Germany (H. Bernard, R. Fischer, M. Wildner); University of Bielefeld, Bielefeld, Germany (R.T. Mikolajczyk); National Institute for Public Health and the Environment, Bilthoven, the Netherlands (M. Kretzschmar); University Medical Center Utrecht, Utrecht, the Netherlands (M. Kretzschmar).

**Keywords:** Influenza, communicable diseases, disease outbreaks, computer simulation, health care sector, nurses, social contacts, viruses, expedited, research

## Abstract

Nurses’ contacts with potentially infectious persons probably place them at higher risk than the general population for infectious diseases. During an influenza pandemic, illness among nurses might result in staff shortage. We aimed to show the value of individual data from the healthcare sector for mathematical modeling of infectious disease transmission. Using a paper diary approach, we compared nurses’ daily contacts (2-way conversation with >2 words or skin-to-skin contact) with those of matched controls from a representative population survey. Nurses (n = 129) reported a median of 40 contacts (85% work related), and controls (n = 129) reported 12 contacts (33% work related). For nurses, 51% of work-related contacts were with patients (74% involving skin-to-skin contact, and 63% lasted <15 minutes); 40% were with staff members (29% and 36%, respectively). Our data, used with simulation models, can help predict staff availability and provide information for pandemic preparedness planning.

## Abstract 2

These data can help predict staff availability and provide information for pandemic preparedness planning.

During past influenza epidemics, hospital staff have been confronted with a surge of inpatients (*1*–*5*), and modeling studies predict collapse of the healthcare system if resources are not allocated carefully (*6*,*7*). To ensure the availability of healthcare during a pandemic, maintaining qualified staff capacity is crucial. Some pandemic preparedness plans therefore prioritize healthcare workers (HCWs) for preventive interventions, such as prophylaxis with antiviral drugs or vaccination (*8*–*10*). In addition to their indispensability, HCWs most likely are at higher risk than the general public for influenza because of their close interaction with infected patients and, presumably, more overall contacts.

Some simulation models use data on social contacts to account for disease spread (*11*–*15*). Models also have been used to assess the effectiveness of vaccinating HCWs against influenza in nursing homes (*16*). Several recently published studies reported contact rates for different population subgroups (*17*–*22*) and even representative population samples from different countries (*23*–*25*), but HCWs’ contacts were not explicitly assessed. This lack of information curtails planning for pandemic preparedness. The current approach to identifying critical threshold parameters for a pandemic is to model disease spread in the population. However, the focus on the general population and an uncritical generalization of model parameters could potentially bias the assessment of disease spread and of available staff capacity within the healthcare sector. We aimed, therefore, to compare social contact data from nurses with data from matched controls in the general population.

## Methods

We conducted a prospective contact survey of nurses in charge of inpatient care for 5 hospitals in the German federal state of Bavaria by using a paper diary approach. We compared the data with data from a matched sample of the German general population obtained in a previously conducted survey of contacts (*25*). In both surveys, a contact was defined as a 2-way conversation of >2 words (not by telephone) or skin-to-skin contact (*17*,*18*) as a surrogate for exposure to disease. Actual rates of disease transmission were not measured.

### Study Population

#### Nurse Sample

Hospitals in Germany are classified by level of care as basic, intermediate, and maximum. We selected 5 hospitals and 5 substitute hospitals representing the distribution of available hospital beds in different Bavarian regions and across the levels of care (*26*). If the head of a hospital refused consent for participation, we approached the head of a substitute hospital.

We included only nurses from the departments of internal medicine or surgery because these departments provide the majority of hospital beds in Germany. We assumed that during an influenza pandemic, most hospitalized persons would be admitted to these departments.

To equally represent work shifts (morning, afternoon, and night) and days of the week, we assigned combinations of work shifts and days for data collection to each hospital and department. Before the survey began, we visited the study hospitals and briefed the nurses on the study aims and methods. We asked the heads of the nursing departments to randomly select 1 of the nurses on duty during each assigned work shift on the assigned day. If the selected nurse refused to participate, another nurse from the same shift was randomly chosen. All nurses provided written informed consent before participating in the study.

We assumed a dropout rate of 20% of initial study participants and accounted for that proportion of declining participants. The calculated sample size for a normally distributed variable with a type I error of 5%, a type II error of 20%, and a difference of 5 in the mean number of contacts between nurses and an equally sized control group (SD = 15 in both groups) was adapted for nonparametric testing by a 15% increase (*27*). Hence, the estimated sample size for our study was 160 participants.

#### Matched Controls

We matched 1 control to each nurse by age (±3 years), sex, and day of data collection (Monday–Friday vs. the weekend [i.e., Saturday–Sunday]). Controls, who were not necessarily from Bavaria, originated from a contact survey of the German population conducted as part of the Improving Public Health Policy in Europe through the Modeling and Economic Evaluation of Interventions for the Control of Infectious Diseases (POLYMOD) project, which is described elsewhere (*25*). In brief, survey participants were recruited by an independent market research company. A representative household sample was selected by using a random-walk technique. In each household, the person with the birthday nearest the date was interviewed. After a face-to-face interview (multitheme survey), respondents filled out a contact diary during the following day. The diaries were collected in person. No incentives were given. We restricted the sample used for comparison with nurses to the summer round of the survey (May–July 2006) to correspond with the period of the survey of nurses. This subsample comprised 340 participants.

### Data Collection

We used a modified version of both the questionnaire and the contact diary designed for the POLYMOD contact survey (*25*). We collected sociodemographic information about each study participant. Participants were asked to complete the diary during a 24-hour period starting at 5 am on the assigned day. The diary was organized as a table in which participants recorded the following features of their contacts during work and leisure time: age (or age range) and sex of the contact person; location where the contact occurred (multiple locations possible); indication of whether physical contact was involved; and length of time the contact lasted. For each contact person, 1 row of the table had to be completed. If a participant had repeated contact with 1 person during the 24 hours of data collection, the characteristics of the contacts with this person were summarized. Additionally, we asked nurses to specify the contact person at work (patient, hospital staff, or other [e.g., visitor]) and, for contacts during travel, means of transportation (public or private).

If controls estimated their total number of contact persons at work to be >10, they were asked to report them aggregated, without specifying contact duration and other characteristics. If controls completed the diary on Monday–Friday, the aggregated contacts were added to those reported in the diary. Therefore, duration and other characteristics of controls’ work contacts were not always available for analysis.

### Data Analysis

We compared median number of contacts by using the Wilcoxon rank sum test or the Kruskal-Wallis 1-way analysis of variance. To compare numbers of contacts for nurses and controls, we used the Wilcoxon signed rank test for matched pairs. These analyses were completed with Stata 10.0 (StataCorp, College Station, TX, USA). In addition, to compare age mixing matrices, we grouped nurses, controls, and the persons they contacted by age and calculated mean number of contacts for each age group.

## Results

### Nurses

We selected 2 hospitals providing basic care, 1 providing intermediate care, and 2 providing maximum care. We replaced 1 of the selected hospitals providing maximum care because the head of the hospital did not consent to study participation. A total of 131 (82% response) nurses completed the diary during April–July 2007.

Nurses reported a median of 40 contacts (range 12–80) during 24 hours (Table 1). Total numbers of contacts did not differ by nurses’ sex or by hospital department (surgery or internal medicine) or level of care. However, nurses had more contacts during Monday–Friday than during the weekend. A median of 34 contacts (range 3–66) were work related.

**Table 1:** Characteristics of 131 nurses surveyed to determine extent of work and other contacts, Bavaria, Germany, April–July 2007*

Characteristic	No. (%) nurses	Median no. reported contacts	p value
Sex			
Female	115 (88)	40	0.35
Male	16 (12)	41	
Leisure activities >1×/wk in group of >5 persons	68 (52)		
Use of public transportation			
Any	86 (66)		
Daily	7 (8)		
Day of diary completion			
Weekday (Monday–Friday)	96 (73)	41.5	<0.05
Weekend (Saturday/Sunday)	33 (25)	32	
Unknown	2 (2)		
Hospital department			
Internal medicine	60 (46)	40	0.46
Surgery	60 (46)	41.5	
Both	11 (8)		
Hospital level of care			
I (basic)	60 (46)	42	0.35
II (intermediate)	20 (15)	31.5	
III (maximum)	51 (39)	39	

*Mean age of nurses was 35 y (range 18–59 y). Mean number of household members was 2 (range 1–7).

Nurses reported a total of 5,161 contacts. Most took place at work, more than half lasted <15 minutes, more than half involved skin-to-skin contact, and more than one third were with persons >60 years of age (Table 2). Ages of contact persons differed for work compared with other places. At work, most contacts were with persons >60 years of age; a small percentage was with persons <20 years. At other places, most contacts were with persons 20–39 years of age, and the proportion with persons <20 years was higher than the proportion at work.

**Table 2:** Characteristics of 5,161 contacts reported by surveyed nurses, Bavaria, Germany, April–July 2007

Characteristic	Reported contacts, no. (%)
Place of contact	
Work	4,207 (82)
Home	226 (4)
Transportation	32 (<1)
Other places	349 (7)
Multiple locations	232 (4)
Not specified	115 (2)
Contact duration	
<15 minutes	2,650 (51)
>15–60 minutes	1,244 (24)
>1 hour	1,202 (23)
Not specified	65 (1)
Contact intensity	
Skin-to-skin	2,646 (51)
Not skin-to-skin	2,331 (45)
Not specified	184 (4)
Age of contact person, y	
<20	284 (6)
20–39	1,535 (30)
40–59	1,558 (30)
>60	1,776 (34)
Not specified	8 (<1)
Age of person contacted at work, y*	
<20	160 (4)
20–39	1,171 (28)
40–59	1,264 (30)
>60	1,611 (38)
Not specified	1 (<1)
Age of person contacted outside work, y†	
<20	94 (15)
20–39	226 (37)
40–59	204 (34)
>60	83 (14)

*n = 4,207. †n = 607.

Most work-related contacts were with patients (51%) or other staff members (40%); 9% were with other persons. Of those with patients, 74% involved skin-to-skin contact, and 63% lasted <15 minutes. Of contacts with staff members, 67% were nonphysical, and 62% lasted >15 minutes. Most contacts with other persons were nonphysical (79%) and lasted <15 minutes (87%) (Table 3).

**Table 3:** Work-related contacts of nurses, Bavaria, Germany, April–July 2007*

Contact duration, min	No. nonphysical contacts with					No. of physical contacts with			
	Patients	Staff	Other	All		Patients	Staff	Other	All
<15	441	507	279	1,227		927	114	37	1,078
15–60	69	332	12	413		483	135	10	628
>60	7	319	5	331		226	247	17	490
Total	517	1,158	296	1,971		1,636	496	64	2,196

*Only contacts with information on all 3 variables (n = 4,167).

### Matched Comparison

We matched controls to 129 (98%) nurses; 2 nurses could not be matched because day of data collection was unknown. Twelve percent of controls were housewives or were unemployed or retired.

Matched nurses reported more total contacts than did controls (5,071 vs. 2,741; median 40 vs. 12; p<0.0001) and more contacts at work (4,288 vs. 1,996; median 34 vs. 4; p<0.0001) (Figure). In other locations, numbers of contacts were similar (783 vs. 745; both medians 5, p = 0.73). In both samples, more contacts occurred during Monday–Friday than during the weekend. Regardless of day of data collection (Monday–Friday or weekend), nurses had more contacts than did controls (Monday–Friday: total contacts median 41.5 vs. 21, p<0.0001; work median 35.5 vs. 16, p<0.0001; other, both medians 5, p = 0.79; on weekends: total contacts median 32 vs. 6, p<0.0001; work median 27 vs. 0, p<0.0001; other median 5 vs. 4, p = 0.85).

**Figure:**
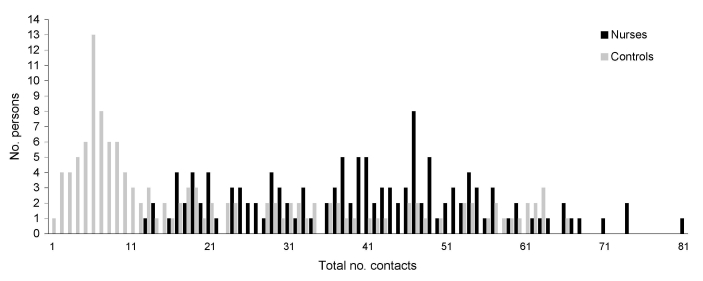
Total numbers of contacts for surveyed nurses and their matched controls from the general population, Bavaria, Germany, April–July 2007.

We calculated mean number of individually reported contacts by nurses’ ages or controls’ ages and by contact persons’ ages (excluding those at work reported by controls only in aggregated manner) (Table 4). Whereas controls tended to interact with persons from their own age group, nurses interacted with persons from a wider variety of age groups, primarily because of the inclusion of older age groups among patients.

**Table 4:** Mean number of individually reported contacts between nurses or controls and other persons, by age group, Bavaria, Germany, April–July 2007*

Age group, y	No. nurses/ controls	Mean no. contacts by age group of contact person									
		0–4 y	5–14 y	15–24 y	25–34 y	35–44 y	45–54 y	55–64 y	65–74 y	75–84 y	85–94 y
Nurses											
15–24	32	0.00	0.13	2.91	4.09	2.94	2.53	2.22	3.00	2.88	0.81
25–34	29	0.28	0.55	6.21	9.31	9.69	7.24	6.79	5.97	5.17	1.45
35–44	31	0.29	1.03	4.13	4.74	6.16	6.13	3.68	5.81	4.94	1.23
45–54	30	0.30	0.63	3.47	5.03	6.40	5.93	3.90	3.87	2.83	1.13
55–64	7	0.29	0.29	2.71	4.57	5.43	3.29	3.43	4.43	3.86	0.43
Controls											
15–24	32	0.00	0.29	6.10	2.38	1.43	1.71	0.76	0.14	0.14	0.00
25–34	29	0.29	0.45	0.60	1.48	1.17	0.74	0.64	0.14	0.07	0.02
35–44	31	0.13	0.26	1.29	1.00	2.29	1.48	0.52	0.42	0.26	0.00
45–54	30	0.07	0.18	0.86	0.96	1.61	1.93	0.71	0.36	0.25	0.00
55–64	7	0.00	0.57	0.57	1.43	0.71	1.86	1.71	0.43	0.14	0.00

*Total number of contacts was 5,071 for nurses and 993 for controls. Controls were not all from Bavaria.

## Discussion

Our findings correspond with the nature of contact between HCWs and patients. Nurses have a high frequency of close contact with patients, but the time they can dedicate to each patient is limited. This pattern differed fundamentally from that of their work contacts with staff members or other persons, the characteristics of which agreed with those described by Mossong et al. (*25*), who found that contact intensity (i.e., whether physical contact was involved) correlated positively with contact duration in a general population sample.

Contact data are important for modeling the spread of infectious diseases. Our results show that the use of general population data might lead to inaccurate modeling results for the healthcare sector because HCWs’ patterns of contact with patients differ fundamentally from the day-to-day contacts of the general population. However, although contact data form the basis of simulation models, other parameters may modify the influence of contact patterns. Our data might change current modeling predictions with respect to 1) spread of disease within the nurse population that results in staff shortage and 2) spread of disease from nurses to the general population.

Our findings suggest that the risk for infectious diseases by airborne transmission might be greater for nurses than for the general population because of nurses’ more frequent and more intensive professional contacts with potentially infected patients. In addition, nurses’ high proportion of work-related skin-to-skin contact highlights the potential for fecal–oral transmission of disease (e.g., norovirus infection).

Whether increased risk for infection in nurses during an influenza pandemic would lead to an earlier peak in the number of infections in HCWs than in the general population needs to be investigated in modeling studies. During a pandemic, maintaining sufficient staff during peak hospitalizations of the general population will be crucial. Our data may help guide public health interventions to prevent the infections in hospital staff and in the general population from peaking simultaneously.

Nurses most likely would take preventive measures, such as wearing personal protective equipment, when handling patients with clinically manifested influenza. However, their risk for infection from patients hospitalized for other reasons and already infected with influenza, but not yet clinically ill or with influenza diagnosis, might be more important for initial spread of disease in the healthcare sector. We collected our data during the interpandemic period, so we cannot predict how HCWs’ contact behavior might change during a pandemic. Models using our data will need to account for this uncertainty.

Nurses can potentially spread infection from infected patients to the general population. In Germany, nurses represent 0.5% of the population (*28*). After infection is introduced into the nurse population, further contact with patients, other staff members, and relatives might result in faster spread to the general population during the early phase of a pandemic. The combination of contact with persons from older age groups at work and from younger age groups at home might facilitate spread among different age groups that would not otherwise occur.

These points support the need to add a separate healthcare component to models, i.e., modeling both HCWs and general population by using self-reported contact data instead of assuming similar contact rates for nurses and the general population. Additional data are required for modeling studies to determine the extent to which nurses and patients would respectively benefit from vaccination of HCWs against influenza.

Our contact definition captures only the amount of social contact with other persons. Because we did not measure rates of transmission or disease, use of our data for modeling relies on the assumption that the number of contacts correlates with the amount of exposure to disease.

Regarding the ongoing discussion about the major route of transmission of influenza (*29*,*30*), we might overestimate exposure to disease by counting conversational contacts if influenza is transmitted predominantly by droplet spread. *On the other hand, accounting only for skin-to-skin contact might considerably underestimate exposure because, among other reasons, transmission by contaminated surfaces or fomites would not be included.* To provide data for modeling the spread of influenza and other infectious diseases with various routes of transmission, we dichotomized the data.

Furthermore, for nurses, the distribution of conversational and skin-to-skin contacts differs among contacts with patients and with others. Consequently, different contact matrices should be used for modeling when different transmission probabilities are assumed for conversational and skin-to-skin contacts.

Our study has some limitations. First, our nurse sample might not be entirely random because potential participants could refuse participation. If nurses willing to participate in the study had more contacts than nurses not willing to participate, our results could overestimate nurses’ contact numbers. Second, nurses’ motivation to participate might have been higher than that of controls, resulting in more reported contacts; a reason for nurses’ increased motivation might be our visits to study hospitals to inform nurses about the aims of the study and the potential benefits of participating for their occupation. The method used to recruit controls from the POLYMOD study required conduct of the survey in study participants’ homes. Even though home visits were timed to minimize this possible selection bias, the sampling still might have resulted in a higher proportion of housewives and unemployed and retired persons, all of whom may have fewer contacts than the general population. However, because patients’ needs largely determine the nurses’ number of work contacts and because the numbers of non–work-related contacts were comparable for nurses and controls, we do not expect that a large selection bias accounts for the difference.

A third potential limitation is the difference in work patterns of nurses and controls. Nurses worked on days they were surveyed, even on weekends, and controls might not have had work contacts during some days of diary completion. This difference is regarded, not as bias, but as potentially meaningful information that needs to be considered when modeling nurses’ contacts. Still, the separate analysis by day of the week persistently differed between the groups. The difference in work contacts reported by nurses and controls also might be affected by controls’ reporting of aggregated work contacts instead of single diary entries if they usually had >10 contacts at work. Not considering these aggregated work contacts for weekend days might have underestimated controls’ contact numbers. However, again, the separate comparison by day points to more contacts of nurses than controls. Evidence conflicts about whether disaggregated contact reporting is more complete than aggregated reporting. One study showed higher median contact numbers comparing prospective to retrospective reporting among university students (*22*). By contrast, another group that compared a diary approach with a 1 time-point estimation of contacts decided in favor of aggregated contacts because most diaries were completed retrospectively (*31*). However, most respondents in the POLYMOD sample from Germanyof the POLYMOD study (*25*) stated that they completed the diary during the day and not retrospectively. The large difference in work contacts between nurses and controls is difficult to explain solely by different methods of contact reporting.

Finally, we perhaps missed types of contacts that do not include conversation or physical contact. Missed contacts might include crowds (e.g., during public transportation), which have been discussed in the context of airborne spread of infections (*29*,*30*). However, the average risk associated with a conversation or with physical contact can be reasonably assumed to be substantially higher than just presence in the same room.

Hospital structure and tasks of nurses in charge of inpatient care are identical in the different German federal states. Therefore, we expect our results from hospitals in the German federal state of Bavaria to be representative of all of Germany. However, because structures and standards of care might differ in other countries, our data require country-specific validation. Furthermore, patterns of contact for hospital workers other than nurses in charge of inpatient care (e.g., physicians, technical assistants, cleaning personnel, or nurses in emergency departments) might differ substantially and influence the patterns of infectious disease spread in the hospital. However, because most routine daily patient contacts are with inpatient nurses, we are confident our data reflect the pattern of a large proportion of contacts in German hospitals.

Our survey did not account for length of hospital stay. Repeated contacts with long-term patients might not bear the same risk for infection as contacts with newly admitted patients because long-term patients are less likely to import an infection to the hospital. Future modeling studies might investigate the impact of length of hospital stay on disease spread.

In our study, nurses’ patterns of contact differed from those of the general population. Our findings support the need for explicit modeling of the healthcare sector to assess the spread of epidemics. To this aim, our study provides quantitative estimates of contact patterns. On the basis of results of modeling approaches for the healthcare sector, public health policies should be reassessed and revised as necessary.

## References

[1] Glaser CA, Gilliam S, Thompson WW, Dassey DE, Waterman SH, Saruwatari M, Medical care capacity for influenza outbreaks, Los Angeles. Emerg Infect Dis. 2002;8:569–74. 12023911 10.3201/eid0806.010370 PMC2738491

[2] Andreasen V, Viboud C, Simonsen L. Epidemiologic characterization of the 1918 influenza pandemic summer wave in Copenhagen: implications for pandemic control strategies. J Infect Dis. 2008;197:270–8. 10.1086/524065 18194088 PMC2674012

[3] Gani R, Hughes H, Fleming D, Griffin T, Medlock J, Leach S. Potential impact of antiviral drug use during influenza pandemic. Emerg Infect Dis. 2005;11:1355–62. 16229762 10.3201/eid1109.041344 PMC3371825

[4] Kawana A, Naka G, Fujikura Y, Kato Y, Mizuno Y, Kondo T, Spanish influenza in Japanese armed forces, 1918–1920. Emerg Infect Dis. 2007;13:590–3. 10.3201/eid1304.060615 17553274 PMC2725954

[5] Nguyen-Van-Tam JS, Hampson AW. The epidemiology and clinical impact of pandemic influenza. Vaccine. 2003;21:1762–8. 10.1016/S0264-410X(03)00069-0 12686091

[6] Nap RE, Andriessen MP, Meessen NE, van der Werf TS. Pandemic influenza and hospital resources. Emerg Infect Dis. 2007;13:1714–9. 18217556 10.3201/eid1311.070103 PMC3375786

[7] Nap RE, Andriessen MP, Meessen NE, Miranda DR, van der Werf TS. Pandemic influenza and excess intensive-care workload. Emerg Infect Dis. 2008;14:1518–25. 10.3201/eid1410.080440 18826813 PMC2609860

[8] Straetemans M, Buchholz U, Reiter S, Haas W, Krause G. Prioritization strategies for pandemic influenza vaccine in 27 countries of the European Union and the Global Health Security Action Group: a review. BMC Public Health. 2007;7:236. 10.1186/1471-2458-7-236 17825095 PMC2048949

[9] Mounier-Jack S, Coker RJ. How prepared is Europe for pandemic influenza? Analysis of national plans. Lancet. 2006;367:1405–11. 10.1016/S0140-6736(06)68511-5 16650650

[10] WHO global influenza preparedness plan. Pandemic influenza preparedness and response: a WHO guidance document [cited 2009 Jun 29]. Available from http://www.who.int/csr/disease/influenza/PIPGuidance09.pdf

[11] Longini IM Jr, Nizam A, Xu S, Ungchusak K, Hanshaoworakul W, Cummings DA, Containing pandemic influenza at the source. Science. 2005;309:1083–7. 10.1126/science.1115717 16079251

[12] Ferguson NM, Cummings DA, Fraser C, Cajka JC, Cooley PC, Burke DS. Strategies for mitigating an influenza pandemic. Nature. 2006;442:448–52. 10.1038/nature04795 16642006 PMC7095311

[13] Germann TC, Kadau K, Longini IM Jr, Macken CA. Mitigation strategies for pandemic influenza in the United States. Proc Natl Acad Sci U S A. 2006;103:5935–40. 10.1073/pnas.0601266103 16585506 PMC1458676

[14] Bansal S, Pourbohloul B, Meyers LA. A comparative analysis of influenza vaccination programs. PLoS Med. 2006;3:e387. 10.1371/journal.pmed.0030387 17020406 PMC1584413

[15] Eichner M, Schwehm M, Duerr HP, Brockmann SO. The influenza pandemic preparedness planning tool InfluSim. BMC Infect Dis. 2007;7:17. 10.1186/1471-2334-7-17 17355639 PMC1832202

[16] van den Dool C, Bonten MJ, Hak E, Heijne JC, Wallinga J. The effects of influenza vaccination of health care workers in nursing homes: insights from a mathematical model. PLoS Med. 2008;5:e200. 10.1371/journal.pmed.0050200 18959470 PMC2573905

[17] Mikolajczyk RT, Akmatov MK, Rastin S, Kretzschmar M. Social contacts of school children and the transmission of respiratory-spread pathogens. Epidemiol Infect. 2008;136:813–22. 10.1017/S0950268807009181 17634160 PMC2870867

[18] Edmunds WJ, O’Callaghan CJ, Nokes DJ. Who mixes with whom? A method to determine the contact patterns of adults that may lead to the spread of airborne infections. Proc Biol Sci. 1997;264:949–57. 10.1098/rspb.1997.0131 9263464 PMC1688546

[19] Edmunds WJ, Kafatos G, Wallinga J, Mossong JR. Mixing patterns and the spread of close-contact infectious diseases. Emerg Themes Epidemiol. 2006;3:10. 10.1186/1742-7622-3-10 16907980 PMC1562421

[20] Beutels P, Shkedy Z, Aerts M, Van DP. Social mixing patterns for transmission models of close contact infections: exploring self-evaluation and diary-based data collection through a web-based interface. Epidemiol Infect. 2006;134:1158–66. 10.1017/S0950268806006418 16707031 PMC2870524

[21] Wallinga J, Edmunds WJ, Kretzschmar M. Perspective: human contact patterns and the spread of airborne infectious diseases. Trends Microbiol. 1999;7:372–7. 10.1016/S0966-842X(99)01546-2 10470046

[22] Mikolajczyk RT, Kretzschmar M. Social contact data in the context of disease transmission: prospective and retrospective study designs. Soc Networks. 2008;30:127–35. 10.1016/j.socnet.2007.09.002

[23] Fu Y. Measuring personal networks with daily contacts: a single-item survey question and the contact diary. Soc Networks. 2005;27:169–86. 10.1016/j.socnet.2005.01.008

[24] Wallinga J, Teunis P, Kretzschmar M. Using data on social contacts to estimate age-specific transmission parameters for respiratory-spread infectious agents. Am J Epidemiol. 2006;164:936–44. 10.1093/aje/kwj317 16968863

[25] Mossong J, Hens N, Jit M, Beutels P, Auranen K, Mikolajczyk R, Social contacts and mixing patterns relevant to the spread of infectious diseases. PLoS Med. 2008;5:e74. 10.1371/journal.pmed.0050074 18366252 PMC2270306

[26] Bayerisches Staatsministerium für Arbeit und Sozialordnung. Familie und Frauen—Krankenhausplan des Freistaates Bayern—32. Fortschreibung. Munich (Germany): Bavarian State Ministry of Labor and Social Welfare, Family Affairs and Women; 2007.

[27] Lehmann EL. Nonparametrics: statistical methods based on ranks, revised. San Francisco: Holden-Day; 1975.

[28] Federal Statistical Office Germany [cited 2009 Jul 6]. Available from http://www-genesis.destatis.de/genesis/online/online

[29] Brankston G, Gitterman L, Hirji Z, Lemieux C, Gardam M. Transmission of influenza A in human beings. Lancet Infect Dis. 2007;7:257–65. 10.1016/S1473-3099(07)70029-4 17376383

[30] Tellier R. Review of aerosol transmission of influenza A virus. Emerg Infect Dis. 2006;12:1657–62. 17283614 10.3201/eid1211.060426 PMC3372341

[31] Glass LM, Glass RJ. Social contact networks for the spread of pandemic influenza in children and teenagers. BMC Public Health. 2008;8:61. 10.1186/1471-2458-8-61 18275603 PMC2277389

